# Sphingolipid Metabolism and Signaling in Lung Cancer: A Potential Therapeutic Target

**DOI:** 10.1155/2022/9099612

**Published:** 2022-06-28

**Authors:** Mengmeng Lin, Yingying Li, Shiyuan Wang, Bo Cao, Chunyu Li, Guohui Li

**Affiliations:** National Cancer Center/National Clinical Research Center for Cancer/Cancer Hospital, Chinese Academy of Medical Sciences and Peking Union Medical College, Beijing 100021, China

## Abstract

Sphingolipids are important bioactive lipids that not only play an important role in maintaining the barrier function and fluidity of cell membranes but also regulate multiple processes in cancer development by controlling multiple signaling pathways in the signal transduction network. Dysregulation of sphingolipid metabolism is thought to be one of the most important dysregulated pathways in lung cancer, the most prevalent type of cancer in terms of incidence and mortality worldwide. This article focuses on lung cancer, reviewing the important lipids in sphingolipid metabolism and the related enzymes in relation to lung cancer progression and their effects on the tumor microenvironment and discussing their roles in the diagnosis and treatment of lung cancer.

## 1. Introduction

Lung cancer remains the leading cause of cancer-related death worldwide, accounting for 11.4% of new cancer cases and 18% of cancer deaths according to the GLOBOCAN 2020 report [1]. Sphingolipids, including ceramide (Cer), sphingosine-1-phosphate (S1P), sphingosine (Sph), sphingomyelin, and glycosphingolipids, are widely found in living organisms and are important structural components of cell membranes. As bioactive lipids, sphingolipids can be involved in signal transduction related to various important physiological processes, including growth and apoptosis [2, 3]. The main enzymes regulating sphingolipid metabolism include sphingosine kinases (SphKs), ceramidases, and sphingomyelinases. In the past decade, many studies have demonstrated the role and mechanism of sphingolipid metabolism in cancer signaling. With the cloning of various regulatory proteins and enzymes involved in sphingolipid metabolism and the development of metabolomics and advancements in mass spectrometry, the relationships between sphingolipid metabolism and diseases, especially cancer, have been increasingly studied, and drugs targeting sphingolipid metabolism have emerged. Dysregulation of sphingolipid metabolism is thought to be one of the most important dysregulation pathways in lung cancer, but the exact link remains to be elucidated [4]. This article reviews important lipids in sphingolipid metabolism and related enzymes in relation to lung cancer progression and effects on the tumor microenvironment, in addition to discussing their roles in the diagnosis and treatment of lung cancer, with a view toward providing references for related studies.

## 2. Sphingolipid Structure and Metabolism

**Figure 1:**
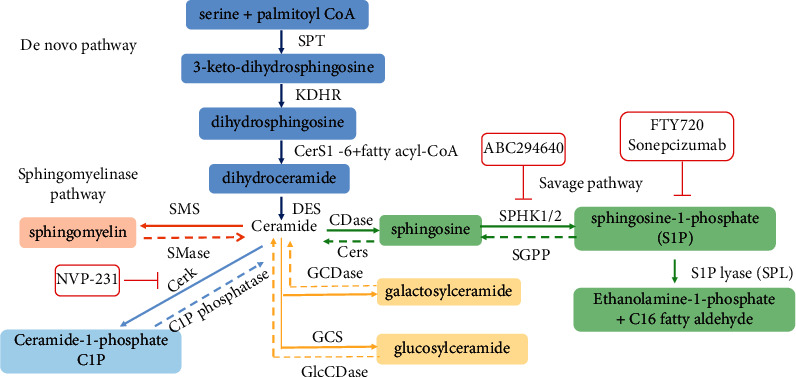
Pathways of sphingolipid metabolism. GCDase, galactosylceramidase; GlcCDase, glucosylceramidase; GCS, glucosylceramide; CerK, synthase ceramide kinase; CerS1-6, ceramide synthase; KDHR, 3-keto-sphinganine reductase; SPT, serine palmitoyltransferase; DES, dihydroceramide desaturase; CDase, ceramidases; CerS, ceramide synthases; SPHK1/2, sphingosine kinase 1/2; SGPP, S1P phosphatase; SMS, sphingomyelin synthase; SMase, sphingomyelinase.

Sphingolipids are amphiphilic lipids composed of sphingosine, fatty acids, and phosphorylcholine. The synthesis of sphingolipids involves *de novo*, savage, and sphingomyelinase pathways (see Figure 1). Because ceramide links the metabolism of multiple sphingolipids, it is often considered central to this metabolic pathway.

Ceramide can be synthesized de novo by the functions of serine palmitoyltransferase (SPT), 3-ketosphinganine reductase (KDHR), (dihydro) ceramide synthases (CERS1–6), and dihydroceramide desaturase (DES). Besides, ceramide can also be generated by the hydrolysis of complex sphingolipids such as sphingomyelin through sphingomyelin pathway. Ceramide is metabolized to sphingosine-1-phosphate (S1P) by the action of sphingosine kinases (SphK1/2) and ceramidases (CDases). Ceramides are often thought to induce proapoptotic effects in cells by modulating multiple targets [5–7]. In contrast, S1P promotes cell survival and proliferation [8]. Previously, researchers have proposed the concept of the “Cer/S1P rheostat,” in which cell death or survival is determined by the balance between ceramide and S1P, with SphKs serving as the key regulatory enzymes of this “rheostat” [9].

## 3. Sphingolipid Metabolism in Lung Cancer Progression

### 3.1. Growth and Proliferation of Lung Cancer Cells

**Figure 2:**
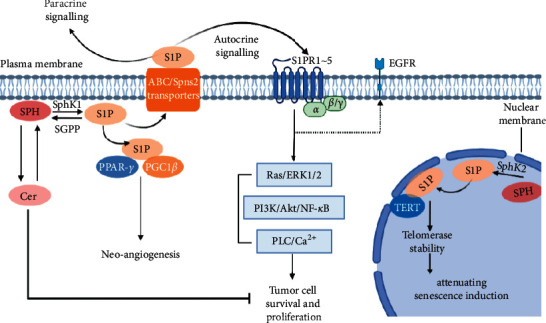
SphK1-generated S1P engages with G protein-coupled S1P receptors (S1PR1–5) to regulate specific cellular functions. S1P also directly associates with PPAR-*γ*, which then mediates the recruitment of PGC1*β* to induce neo-angiogenesis. Generation of S1P by SphK2, which is localized in the nuclear membrane, interacts with TERT to stabilize telomerase and attenuate senescence induction. PI3K, phosphatidylinositol 3-kinase; MAPK, mitogen-activated protein kinases; PLC, phospholipase C; PPAR-*γ*, proliferator-activated receptor-*γ*; TERT, telomerase reverse transcriptase; EGFR, epidermal growth factor receptor; ERK1/2, extracellular signal-regulated kinases 1 and 2; NF-*κ*B, nuclear factor-*κ*B; PLC, phospholipase C; S1P, sphingosine-1-phosphate; S1PR, sphingosine-1-phosphate receptor; Sph, sphingosine; SphK1, sphingosine kinase 1; ABC, ATP-binding cassette; SPNS2, protein spinster homologue 2.

Lung cancer is essentially the uncontrolled growth of malignant lung tissue. As early as the end of the last century, S1P has been suggested to play a key role in cell mitosis [10]. S1P exerts most of its biological effects by regulating downstream pathways through S1PRs, members of the G protein-coupled receptor (GPCR) family, on the cell membrane surface. SphK1 can be activated in the cytoplasm by phosphorylation and translocated to the plasma membrane to produce sphingosine-1-phosphate (S1P) with sphingosine as a substrate. After S1P is secreted by specific transporter proteins, it binds to S1P receptors (S1PR1-5) in an autocrine or paracrine manner and activates downstream signaling pathways such as Ras/ERK1/2 and PI3K/Akt, exerting a variety of biological effects [11]. S1P may also function as an intracellular second messenger by binding to different intracellular partners. In lung adenocarcinoma cells, S1P/S1PR3 was also found to enhance EGFR expression, promote proliferation, and anchor nondependent growth, thereby promoting tumor progression [12] (see Figure 2).

SphKs are key enzymes that regulate the balance between ceramide and S1P. The two main types of SphKs in the human body are SphK1 and SphK2. Upregulation of SphK1 promotes NSCLC cell proliferation and inhibits apoptosis through downstream activation of the PI3K/Akt/NF-*κ*B pathway [13]. It has been shown that SphK1 overexpression induces the expression of antiapoptotic and migration-related genes, including Bcl-2 and matrix metallopeptidase 2 (MMP-2), and promotes the proliferation and migration of NSCLC cells [14]. Further studies revealed that this alteration is mediated by the activation of signal transduction and activator of transcription 3 (STAT3) [14]. In addition, S1P production by SphK2 was found to be associated with the catalytic subunit of telomerase in normal fibroblasts and lung cancer cells, where it promotes cell proliferation and tumor growth [15].

Unlike S1P, ceramide is thought to be an important secondary messenger involved in apoptosis and necrosis pathways in both normal and cancer cells [16, 17]. *In vitro* studies revealed that C2 ceramide can induce apoptosis in lung cancer cells by modulating the TXNIP/Trx1 complex, inhibiting AKT and NF-*κ*B activity, and downregulating survivin and cyclin A2 [18, 19]. In addition, C2 ceramide was shown to significantly affect autophagy-related factors, including SIRT1, LAMP2, and LC3, induce sustained autophagy, and increase apoptosis in non-small cell lung cancer [20].

Ceramide kinase (CerK) phosphorylates ceramide to generate ceramide-1-phosphate (C1P). C1P regulates the growth and survival of A549 cells, and relatively low concentrations of C1P promote the survival of A549 lung cancer cells. Treatment with specific siRNAs to silence the gene encoding this kinase and downregulate CerK expression caused the number of apoptotic lung cells to increase greatly [21]. The CerK inhibitor NVP-231 blocks the M phase of the cell cycle and activates the caspase-9/caspase-3 pathway to promote apoptosis in lung cancer cells, while the overexpression of CerK promotes cell proliferation and protects lung cancer cells from apoptosis [22].

In addition, serine palmitoyltransferase (SPT) mediates the binding of serine and palmitoyl coenzyme A to form ceramide and is thus an important rate-limiting enzyme in sphingolipid metabolism. Osamu et al. [23] showed that the inhibition of SPT resulted in COX-2 overexpression, thereby inducing apoptosis of HCC4006 lung adenocarcinoma cells through a necrosis-dependent pathway. Yaguchi et al. [24] identified a new oral SPT inhibitor that showed antiproliferative effects in several cancer cell models, including lung cancer cells.

### 3.2. Metastases

The aggressiveness of lung cancer cells depends in part on their ability to metastasize. Cell migration is one of the key steps in the invasion and metastasis of malignant tumors, during which filopodium and lamellipodium are overproduced in cancer cells, and the activity of the GTPase signaling complex is highly involved [25, 26]. The activation of the CerK/C1P pathway was found to decrease Rac1-GTP protein expression levels and had an inhibitory effect on lamellipodia formation, migration, and metastasis in A549, HTB177, HTB183, and CRL5803 lung cancer cells [27]. High expression of the ceramide synthase CerS6 is associated with poor lung cancer prognosis and lymph node metastasis [28, 29]. CerS6 knockdown inhibited the formation of Rac1-positive lamellipodia and reduced the efficiency of lung metastasis in mice.

In addition, the ectopic expression of S1PR3 promoted the growth and metastasis of human lung adenocarcinoma cells in mice [30]. Epithelial-mesenchymal transition (EMT) refers to a series of phenotypic changes in which epithelial cells are transformed to acquire mesenchymal properties. EMT-related protein E-cadherin, N-cadherin, and Snail play important roles in the invasion and metastasis of malignant tumor [31]. SphK1 promotes the invasion and migration of NSCLC cells by downregulating E-cadherin protein levels and upregulating Snail protein levels through the AKT pathway [32].

To summarize, metabolic reprogramming is an important marker of tumor formation, and the related enzymes and lipids in sphingolipid metabolism are highly relevant to the growth and proliferation of lung cancer cells. Targeting sphingolipid metabolism, especially the important enzymes involved, may be an important way to inhibit the progression of lung cancer.

## 4. Sphingolipid Metabolism in Tumor Microenvironment

**Figure 3:**
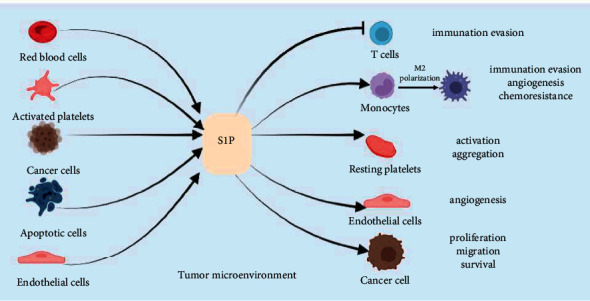
In the tumor microenvironment, red blood cells, activated platelets, cancer cells, apoptotic cells, and vascular endothelial cells can all secrete S1P, which acts on a variety of cells in the tumor microenvironment and produces different effects.

Tumor microenvironment (TME) refers to the surrounding microenvironment in which tumor cells exist, including surrounding blood vessels, immune cells, and platelets. The tumor microenvironment is a complex environment for tumor cell survival and development, and it plays important roles in tumorigenesis and development. In the tumor microenvironment, various cells, including activated platelets, tumor cells, apoptotic cells, and vascular endothelial cells, can produce S1P, which is transported to extracellular areas by transporter proteins and acts on various cells in the tumor microenvironment with various effects (see Figure 3).

### 4.1. Platelets

It has been shown that a large number of platelets are present in the tumor microenvironment, where they play key roles in inducing vascular permeability and promoting tumor metastasis [33]. The relationship between platelets and lung cancer has drawn increasing attention, leading to their consideration as a potential diagnostic tool for lung cancer [34]. Platelets are derived from megakaryocytes in the bone marrow. S1P is involved in the regulation of platelet production as a cellular secondary messenger. In megakaryocytes, S1P regulates platelet production under the control of the expression and activity of Src family kinases, with the deletion of SphK2 suppressing Src family kinase activity and resulting in defective intravascular proplatelet shedding [35]. In addition, it has been shown that S1PR1, S1PR2, and S1PR4 receptors are highly expressed in megakaryocytes and that S1P acts on S1PR1 receptors to activate Gi/Rac GTPase signaling, mediating platelet shedding from preplatelets and promoting their eventual release into circulation [36].

Due to the high activity of SphKs and the lack of S1P lyase, platelets exhibit high levels of S1P [37]. Urtz et al. [38] found that activated state platelets release large amounts of S1P into the blood and that SphK2 is primarily responsible for S1P production in platelets. S1P initiates whole blood aggregation directly through platelet-expressed S1PR1 and promotes platelet aggregation in response to PAR4-P and ADP [38].

In addition to the central role of platelets in the blood coagulation process, tumor-associated platelets have an important function in maintaining tumor vascular integrity in the tumor microenvironment. Platelets are able to maintain the integrity of tumor vascular endothelium and prevent intra-tumor hemorrhage by secreting granule contents such as 5-hydroxytryptamine (5-HT), platelet factor IV (PF-4), and transforming growth factor (TGF)-*β* or by directly adhering to damaged blood vessels [39, 40]. Active sphingolipid metabolism with significantly elevated levels of metabolites, including sphingomyelin (SM) and ceramide, was found in H_2_O_2_-stimulated human platelets [41]. Liu et al. demonstrated that exogenous S1P can alter platelet function in a concentration-dependent manner, with low concentrations of S1P initiating platelet function and high concentrations inhibiting platelet responses [42]. Ceramidase inhibition significantly blunted glycoprotein VI (GPVI)-induced platelet aggregation, which could be partially overcome by exogenous sphingosine [43]. In contrast to the heterogeneity generally displayed by tumor cells, platelets remain relatively unchanged, making antiplatelet therapy a potential and promising avenue for tumor treatment.

The above studies have shown that S1P, an important lipid in sphingolipid metabolism, plays an important role in platelet generation, activation, and aggregation. In contrast to the heterogeneity generally displayed by tumor cells, platelets remain relatively unchanged, making antiplatelet therapy a potential and promising avenue for tumor treatment.

### 4.2. Angiogenesis

Angiogenesis is a key mechanism of tumor growth and is closely related to tumor metastasis and invasion. S1P binds directly to peroxisome proliferator-activated receptor *γ* (PPAR-*γ*), which in turn mediates the recruitment of PPAR-*γ* coactivator 1*β* (PGC1*β*) to induce PPAR-*γ*-dependent gene expression and neovascularization [44]. In the tumor microenvironment, S1P induces macrophages to polarize toward the M2 phenotype and stimulates macrophages to secrete prostaglandin E2 (PGE2), which induces endothelial cell migration and increases angiogenesis [45, 46]. S1P released from apoptotic cells also induces upregulation of Bcl-X (L) and Bcl-2 expression and protects macrophages from cell death [47]. The rapid growth of tumor tissues causes severe local hypoxia, and tumor adaptation to hypoxia is mainly regulated by hypoxia-inducible factors (HIFs), which induce and regulate the expression of angiogenesis-related genes such as vascular endothelial growth factor (VEGF) and Notch. Under hypoxic conditions, SphK1 is activated to stimulate protein kinase B/glycogen synthase kinase 3*β* (Akt/GSK3*β*) signaling via S1P/S1PR2 in a reactive oxygen species (ROS)-dependent manner, regulating HIF-1*α* protein expression levels to promote structurally defective angiogenesis [48, 49].

A variety of cells within the tumor microenvironment, including macrophages, vascular smooth muscle cells, and endothelial cells, produce cytosolic interleukin-8 (IL-8), which activates CXCR1 receptors on endothelial cells to promote angiogenesis [50]. IL-8 levels are upregulated in a variety of cancers, including lung cancer, and S1PR1 signaling induces IL-8 expression, while S1PR2 signaling induces its secretion [51]. In addition, S1P acts on endothelial cells to promote structurally defective angiogenesis, which further promotes tumor cell invasion [52].

Tumor angiogenesis is closely related to the tumor microenvironment and is regulated by several pro-angiogenic factors and/or angiogenic inhibitory factors. These studies suggest that S1P may regulate angiogenesis directly or indirectly by acting on vascular endothelial cells or by promoting the release of angiogenic factors. In recent years, antitumor drugs targeting tumor angiogenesis, such as anlotinib, have benefited many lung cancer patients, yet their inevitable drug resistance remains an urgent problem. Therefore, targeting the relevant enzymes and lipids in sphingolipid metabolism to inhibit angiogenesis may provide a new idea for tumor suppression.

## 5. Sphingolipid Metabolism and Lung Cancer Treatment

### 5.1. Diagnosis

Most lung cancer patients are not diagnosed until advanced or metastatic stages, especially in cases of non-small cell lung cancer (NSCLC). Early diagnosis of NSCLC is a key way to improve prognosis. A population-based cohort nested case-control study showed that geometric mean concentrations of plasma S1P and total ceramide were higher in lung cancer patients than in controls, suggesting these as potential markers of latent lung cancer [53]. Meng et al. [54] analyzed the RNA-seq datasets obtained from TCGA, GEO, and Hou lung and found a consistent alteration of 15 sphingolipid metabolic gene expression in NSCLC patient tissues as compared to the normal lung tissues. Among these genes, the expression of B3GNT5 and GAL3ST1 is most significantly associated with patient prognosis and their metabolites are potential biomarkers for staging patients.

In addition, SphK1, a key regulator of the dynamic balance of Cer/S1P, has been found to be overexpressed in lung cancer tissues [55]. High SphK1 expression is significantly associated with five-year and overall survival rates in cancer, suggesting that SphK1 may be a potential biomarker for predicting prognosis in cancer patients [56].

Immunohistochemical detection of SphK1 expression in non-small cell lung cancer patients receiving platinum-based adjuvant chemotherapy can be a potential indicator for evaluating treatment efficacy [57]. SphK2 levels were also found to be significantly correlated with proliferation index, lymph node status, histological grade, and clinical stage in NSCLC tissues. High SphK2 expression was clearly associated with poor 5-year DFS (27.19 vs. 45.35%) and 5-year OS (31.92 vs. >50%) rate, and all of the normal specimens were SphK2 low or no expression [58].

Ceramide synthases (CerSs), key enzymes of sphingolipid metabolism, have also been considered as potential tumor markers. High expression of CerS2, CerS3, CerS4, and CerS5 was associated with significantly lower OS in patients with NSCLC, while high expression of CerS2 and CerS5 was associated with significantly poorer OS in lung adenocarcinoma [59]. However, conclusions need to be treated with caution because of the lack of high quality of evidence.

### 5.2. Drug Resistance

Drug resistance is extremely common during the treatment of lung cancer and represents one of the major limiting factors in patient care. Intracellular ceramide increases after chemotherapy or radiotherapy, thereby promoting proliferative arrest and apoptosis in tumor cells. The proapoptotic effect of ceramide has been used as an adjuvant to chemotherapy [60]. In sphingolipid metabolism, changes in the levels of enzymes associated with ceramide metabolism can alter intracellular ceramide levels, and a decrease in ceramide levels may lead to the development of drug resistance.

A variety of drug-resistant cells exhibit high levels of SphK1 expression in vitro. For example, in imatinib-resistant chronic myeloid leukemia (CML), SphK1 overexpression induces resistance through S1PR2-mediated activation of PP2A signaling [61]. In NSCLC cells, upregulation of SphK1 significantly inhibits apoptosis induced by adriamycin or doxorubicin through activation of the PI3K/Akt/NF-*κ*B pathway, and the specific inhibitor SK1-I significantly increases the sensitivity of NSCLC cells to chemotherapeutic drug-induced apoptosis by blocking SphK1 expression or inhibiting SphK1 kinase activity [62]. As with SphK1, high SphK2 expression was associated with drug resistance [58]. SphK2 inhibitor (ABC294640) and TRAIL could increase caspase-3/8 activity and death receptor expression levels, thereby promoting apoptosis in non-small cell cancer cells [63].

Glycosphingolipids (GSLs) are a subtype of glycolipids synthesized by ceramide glycosylation. Tumor cells convert ceramide to glucoceramide via glucoceramide synthase (GCS), which further generates GSLs and decreases ceramide content [64]. Conversion of ceramide to glucoceramide by GCS has been shown to be associated with drug resistance in a variety of cancers [65]. One study found that GCS may play a regulatory role in cisplatin resistance in non-small cell lung cancer and mesothelioma tumor cells, and inhibitors of GCS activity significantly reversed this resistance [66].

From the research described above, it is clear that GCS and SphK1/2 are potential therapeutic targets to overcome drug resistance, and the accumulation of glucoceramides and S1P may also serve as potential predictive biomarkers of chemotherapy resistance in various cancers.

### 5.3. Therapeutic Targeting of Sphingolipids

In recent years, there has been a gradual increase in the number of studies treating lung cancer by modulating sphingolipid metabolism, with most still in preclinical stages (see Table 1). These efforts primarily include targeting S1P receptors, inhibiting SphK activity, and treatment with S1P-specific antibodies.

#### 5.3.1. FTY720

FTY720/fingolimod is a recently developed immunosuppressant modified from ISP-I, an immunosuppressive component of *Cordyceps sinensis* extract, and has been successfully used to treat relapsing multiple sclerosis by targeting sphingolipid signaling [67]. FTY720 can be phosphorylated by SPHK2 *in vivo* to generate PFTY720, a structural analogue of S1P, which acts as an antagonist of S1PR1 and can inhibit the growth of colon and lung cancer cell lines through S1PR receptor-dependent and non-receptor-dependent mechanisms. The combination of FTY720 and cisplatin inhibited the growth of A549 cells more effectively than drug treatment alone and enhanced *in vivo* antitumor activity in mice with lung cancer [68]. Booth et al. reported that FTY720 enhances the killing effect of pemetrexed in non-small cell lung cancer and overcomes resistance to ERBB inhibitors [69].

#### 5.3.2. ABC294640

Many cytokines and antitumor drugs increase endogenous ceramide content and inhibit S1P production by inhibiting the *de novo* pathway or sphingomyelin degradation, thus exerting antitumor effects [70–72]. ABC294640 is a selective inhibitor of both SphK2 and DES1 that decreases S1P synthesis. *In vitro*, ABC29460 has been shown to reduce the proliferation and survival of transplanted tumors in a variety of cancer cell lines in mice while exhibiting low levels of toxic effects [67, 72, 73]. ABC294640 treatment led to significant apoptosis, cell cycle arrest, and tumor growth inhibition in NSCLC cells *in vitro* and *in vivo*, and ABC29460 treatment was found to alter the composition of ceramide and dihydroceramide in NSCLC [74]. Guan et al. found that inhibition or silencing of glucoceramide synthase (GCS) caused further accumulation of ceramide by ABC294640 and promoted apoptosis of cancer cells, suggesting that the combination of ABC294640 with a GCS inhibitor may have better anticancer effects [75].

In addition to what was mentioned above, miR-338-3p inhibits NSCLC cell proliferation and induces apoptosis by targeting and downregulating SphK2 [76]. NVP-231, an inhibitor of CerK, decreases cell survival in a concentration-dependent manner in the lung cancer cell line NCI-H358 and causes cellular M-phase arrest [22].

Currently, most research regarding the treatment of lung cancer by targeting sphingolipid metabolism is at the preclinical stage. Although drugs targeting enzymes or receptors in the sphingolipid metabolism process have entered clinical studies for some cancer types, problems such as side effects and poor clinical outcomes have greatly limited further development. In addition, even for a single target, significant differences still exist between different cancer types. Therefore, even though the importance of targeting sphingolipid metabolism in cancer has become appreciated by researchers in recent years, further research is still needed for lung cancer, an important cancer type. For example, enhanced drug targeting and further elucidation of mechanisms of action are important for future study of related drugs.

In summary, various enzymes involved in sphingolipid metabolisms such as SphKs and CerSs can be used as potential biomarkers for the diagnosis of lung cancer and determination of disease progression. High expression of SphKs is closely related to acquired drug resistance in lung cancer patients, and specific inhibition of related enzymes may improve the inhibitory effect of chemotherapeutic drugs on lung cancer cells. Studies targeting sphingolipid metabolism to inhibit lung cancer are also gradually being conducted, with some achieving good results in preclinical studies. Further studies on the molecular biology of sphingolipid metabolism in lung cancer progression and to investigate whether sphingolipid metabolism-related enzymes and lipids can be used as a reliable biomarker for lung cancer diagnosis and disease progression in a broader context are important for improving lung cancer treatment and early lung cancer screening.

## 6. Summary and Prospects

There have been many discoveries elucidating the roles and mechanisms of sphingolipids in cancer signaling during the past decade. Sphingolipids are not only important components of biological membranes but also have various biological activities. Sphingolipid metabolism involves a variety of metabolites and enzymes, which play important roles in lung cancer growth, proliferation, and metastasis. Dysregulation of sphingolipid metabolism is considered to be one of the most important pathways of metabolic dysregulation in lung cancer patients. The study of relevant biomarkers may provide a reliable method for early screening of lung cancer. In addition, the roles of sphingolipid metabolism in influencing the tumor microenvironment require further attention, which will help to develop novel therapeutic strategies to inhibit cancer growth, proliferation, and metastasis.

Ceramide and S1P are two key sphingolipid molecules that have been investigated extensively. Their different subcellular localization patterns and downstream targets underlie their opposing functions, but their specific mechanisms of action remain to be investigated due to the presence of complex regulatory mechanisms, including multiple ceramide isoforms. The role of S1P in cancer is not limited to enhance tumor growth and metastasis, but S1P is emerging as a key signal in the regulation of communication between tumors and host cells associated with the tumor microenvironment.

At present, although there are corresponding sphingolipid-targeted drugs in clinical trials, and many preclinical studies have shown that sphingolipid-targeted drugs have better efficacy in combination with other therapeutic modalities, such as immunotherapy or conventional chemotherapy, few drug designs or studies target sphingolipid metabolism in lung cancer, and further studies are needed to enhance the ability of targeting treatment and reduce side effects. In conclusion, advancing the understanding of the relationship between sphingolipid metabolism and lung cancer will help to illuminate the pathogenesis of lung cancer, develop new therapeutic strategies, and overcome drug resistance.

## Figures and Tables

**Table 1:** List of anti-lung cancer agents targeting sphingolipid metabolism.

Name	Target or activity	Cell type	Function	Refs
FTY720	S1PR1; I2PP2A	A549 lung adenocarcinoma cells	Inhibitor	[68, 77]
ABC294640	SphK2; DES	NSCLC cell lines	Inhibitor	[74]
Myriocin	SPT	Human lung adenocarcinoma cell line (HCC4006);	Inhibitor	[23]
N-((3S,4R)-1-((8-chloroquinoxalin-6-yl)carbonyl)-3-phenylpiperidin-4-yl)-1-methyl-3-(trifluoromethyl)-1H-pyrazole-5-carboxamide	SPT	A549 lung adenocarcinoma cells; human lung adenocarcinoma cell line (HCC4006); NCI-H460 human lung cancer; NCI-H522 human lung cancer	Inhibitor	[24]
Tetrahydropyrazolopyridine; 3-phenylpiperidine	SPT	Human lung adenocarcinoma cell line (HCC4006)	Inhibitors	[78]
Antifolate methotrexate	CesS	A549 lung adenocarcinoma cells	Indirect activator	[79]
N, N-Dimethyl-D-erythro-sphingosine (DMS)	SphK1	A549 lung adenocarcinoma cells	Inhibitor	[80]
Ellagic acid	SphK1	A549 lung adenocarcinoma cells	Inhibitor	[81]
Harmaline	SphK1	A549 lung adenocarcinoma cells	Inhibitor	[82]
PGR260	SphK1	A549 and NCI-H1944 cell lines	Inhibitor	[83]
GDC-0349	SphK1	A549 lung adenocarcinoma cells	Inhibitor	[84]
miR-338-3p	SphK2	A549 and H1299 cell lines	Downregulation	[76]
NVP-231	CerK	NCI-H358 human lung cancer cell lines	Inhibitor	[22]

## Data Availability

The data used to support the findings of this study are available from the corresponding author upon request.
